# Rethinking Advanced Renal Cell Carcinoma: Integrative Genomics, Immunotherapy, and Molecular–Orthomolecular Strategies

**DOI:** 10.3390/cancers18091435

**Published:** 2026-04-30

**Authors:** Marijana Turčić, Kristian Krpina, Dragan Trivanović, Krešimir Pavelić, Sandra Kraljević Pavelić

**Affiliations:** 1 Teaching Institute of Public Health of Primorsko-Goranska County, Krešimirova 52a, 51000 Rijeka, Croatia; turcic.m.ri@gmail.com; 2 Department of Urology, Clinical Hospital Center Rijeka, Tome Strižića 3, 51000 Rijeka, Croatia; kristian.krpina@medri.uniri.hr; 3 Department of Urology, Faculty of Medicine, University of Rijeka, Braće Branchetta 20, 51000 Rijeka, Croatia; 4 Department of Oncology and Hematology, General Hospital Pula, Santorijeva 24a, 52200 Pula, Croatia; dragan.trivanovic@obpula.hr; 5 Faculty of Medicine, Juraj Dobrila University of Pula, Zagrebačka 30, 52100 Pula, Croatia; pavelic@unipu.hr; 6 International Academy of Science and Arts in Bosnia and Herzegovina, Radnička cesta, 71000 Sarajevo, Bosnia and Herzegovina; 7 Department of Basic Medical Sciences, Faculty of Health Studies, University of Rijeka, Viktora Cara Emina 5, 51000 Rijeka, Croatia

**Keywords:** renal cell carcinoma, genomic fusion biology, cell-based immunotherapy, molecular targeting, orthomolecular therapeutic approaches

## Abstract

Renal cell carcinoma (RCC) is acknowledged as a heterogeneous malignancy underlined by complex genetic, metabolic, and immune dysregulation. In particular, molecular studies have revealed distinct oncogenic mechanisms that have been exploited and studied as therapeutic intervention targets. These include hypoxia-driven signaling, chromosomal translocations, and gene fusion events that affect tumor progression. This review provides a comprehensive overview of these targets and rethinks RCC management. Therapeutic concepts include the targeting of genomic fusion biology with emerging cell-based immunotherapies or targeted molecular inhibition, and orthomolecular therapeutic strategies are presented. Two clinical and pathological features are highlighted—namely, the TFE3 fusion proteins in translocation RCC and the growing role of hypoxia-inducible factor-2α (HIF-2α) inhibitors in clear-cell RCC. We also present recent data on novel immunotherapeutic approaches, including autologous hematopoietic stem and progenitor cell-based interferon-α gene therapy, as well as chimeric antigen receptor T-cell therapy. These therapies are discussed in light of their mechanistic rationale, translational potential, and existing clinical challenges due to unwanted side effects. At last, orthomolecular and natural product-based therapies are reviewed for their potential as adjunctive therapies that might be used for oxidative stress management, the targeting of tumor metabolism and immune effects, and to increase standard treatment tolerance. This review points to a multidimensional framework that might support further research and studies in precision-guided RCC management, as integrative approaches may enhance therapeutic efficacy, reduce toxicity, and support the development of personalized interventions for advanced or treatment-resistant RCC.

## Simple Summary

Renal cell carcinoma (RCC) is a complex disease driven by genetic alterations, metabolic changes, and immune-system interactions. In recent decades, advances in molecular research have revealed key mechanisms in RCC development. These mechanisms have been studied as potential targets for therapeutical intervention and include hypoxia-related signaling pathways and gene fusion events. This review accordingly summarizes current therapy and presents emerging treatment strategies for advanced RCC, with particular focus on TFE3 fusion proteins in translocation RCC and hypoxia-inducible factor-2α (HIF-2α) inhibitors in clear-cell RCC. Novel immunotherapeutic approaches are also discussed, especially those based on chimeric antigen receptor (CAR) T-cell therapy and autologous stem cell-based interferon-α gene therapy. At last, the review explores the possibility of orthomolecular and natural product-based strategies as supportive therapies that show pre-clinical potential in the reduction of oxidative stress and modulation of tumor metabolism while showing promise in terms of increasing treatment tolerance. This work emphasizes the importance of integrative and precision-based approaches in improving outcomes for patients with advanced or treatment-resistant RCC.

## 1. Introduction

Cancer development is described as a dynamic and multistep process. It is characterized by cell-level changes that include sustained proliferative signaling, resistance to cell death, metabolic reprogramming, immune evasion, and the occurrence of angiogenesis and metastases. These are main hallmarks of cancer that have been used by researchers as a conceptual framework for understanding tumor behavior and the development of therapeutic strategies. Briefly, cancer treatment has evolved in recent decades from non-specific cytotoxic approaches towards mechanism-based immunotherapies. The latter have been particularly studied due to an improved understanding of tumor biology and its interaction with the host immune system. Contemporary oncology, in particular, emphasizes integrative strategies that take into account known molecular drivers of disease [1]. The emerging conceptual linkage between cancer biology and therapeutic evolution is particularly relevant in tumors with distinct molecular and immunological features. This is particularly true for renal cell carcinoma (RCC).

Localized renal cell carcinoma (RCC) is the most common malignancy of the urinary tract and is acknowledged as a curable disease; patients have a 5-year overall survival rate of approximately 93%. A detailed classification of RCC is given in Table 1. RCC is a biologically distinct entity among solid tumors. RCC has been studied in detail, particularly with respect to its metabolic alterations, highly vascular phenotype, and high immunogenicity. These characteristics arise, in particular, in association with disruptions in oxygen-sensing pathways, chromatin remodeling, and cellular metabolism. RCC progression is accordingly driven by these collective features that have also been exploited as therapeutic vulnerabilities.

The markers of the heterogeneity of RCC, such as the VHL/HIF axis in clear-cell RCC, MET signaling in papillary RCC, and metabolic enzyme deficiencies (e.g., FH and SDH) in rare but aggressive variants, give rise to diverse histological subtypes and underlying molecular alterations. This is why RCC presents with differences in clinical behavior, metastatic potential, and treatment response. The main molecular features provide a strong rationale for the development of targeted and immune-based therapies.

Still, metastatic RCC has a substantially poorer outcome, with a 5-year survival rate of around 18% [33]. These data were assessed primarily for the most common histological subtype, clear-cell RCC. The most common symptoms of RCC are a palpable flank mass, hematuria, pain, and weight loss, but in everyday practice, the majority of RCC patients are asymptomatic and identified by chance. The most common metastatic sites for advanced RCC are the lungs, liver, lymph nodes, adrenal glands, and brain [34]. From a therapeutic perspective, the RCC paradigm clearly shows a shift from empiric to biology-driven oncology. For example, cytokine-based therapies such as interferon-gamma ae limited by modest efficacy and substantial toxicity. This is a consequence of an incomplete understanding of RCC mechanisms. In contrast, targeted therapies and immune checkpoint inhibitors directly exploit RCC-specific vulnerabilities. Cytoreductive nephrectomy is still a key approach in disease management, even in cases presenting with metastatic disease at diagnosis, as it may be associated with improved survival outcomes [35,36]. In particular, RCC is a highly immunogenic tumor such as, for example, melanoma and non-small-cell lung cancer. This is relevant from a therapeutic perspective, as immunotherapy in this group of tumors has demonstrated significant clinical benefit [37]. In particular, immune checkpoint inhibitors (ICIs) and their combinations revolutionized the treatment of advanced and metastatic RCC, which led to substantial survival improvements. Proven ICIs target programmed cell death protein 1 (PD-1), programmed cell death ligand 1 (PD-L1) or cytotoxic T lymphocyte-associated protein 4 (CTLA-4). Nevertheless, this field is now developing into novel checkpoint agents, i.e., those targeting immunoglobulin and mucin domain 3 (TIM-3), lymphocyte activating gene 3 (LAG-3) or Ig V domain suppressor for T cell activation (VISTA), which have been studied for application in combination with other ICIs to target multiple pathways and immune checkpoints [38]. This treatment approach is now the first-line treatment standard. Indeed, dual immunotherapy regimens and combinations of ICIs that particularly enhance antitumor immune responses by blocking co-inhibitory signaling pathways within the tumor microenvironment, along with tyrosine kinase inhibitors (TKIs), have shown overall survival benefits in previously untreated patients [39,40,41,42]. However, immune-related adverse events have been observed with these therapies. These include the heightened immune activation that targets healthy tissues, posing significant clinical management challenges [43]. Early identification of adverse prognostic factors is critical to determine which patients may benefit from the adjuvant therapy. Previous therapy regimens relied on the use of interferon-gamma but were largely ineffective due to limited efficacy and high levels of toxicity [44]. Historically, the lack of reliable biomarkers for evaluation of possible treatment approaches necessitated reliance on clinical prognostic models that have often exhibited limited predictive performance [45,46,47]. In recent years, accordingly, researchers have worked on the integration of clinical parameters with multi-omics approaches, all supported by artificial intelligence, as a promising strategy to enhance prognostic accuracy and guide treatment personalization [48]. This is relevant for the assessment of the need for immunotherapy and choice of treatment combinations. For example, recent multi-omics studies have provided detailed insights into molecular subtypes of RCC that have prognostic and predictive relevance. These analyses have identified the so-called “immune hot” and “immune cold” tumor profiles, B-cell-associated gene signatures, and tumor stemness markers. These identified signatures may influence the response to immunotherapy [37,39]. Furthermore, high-throughput proteomic studies, such as the Olink Explore platform in the IMmotion010 trial, revealed Kidney Injury Molecule-1 (KIM-1) as a potentially predictive biomarker in RCC. This highlights its translational relevance for early detection of recurrence and immunotherapy stratification [49,50]. Adjuvant immunotherapy has recently demonstrated meaningful clinical benefit in high-risk RCC following surgical resection. Indeed, several clinical trials support the value of immunotherapy for prevention of recurrence. These include trials evaluating atezolizumab (IMmotion010), nivolumab plus ipilimumab (CheckMate 914), and pembrolizumab (KEYNOTE-564) [51,52,53,54]. Moreover, spatial analyses and single-cell profiling may be used to enhanced understanding of the tumor immune architecture and mechanistic insights to inform therapeutic strategies discussed in subsequent chapters [37,39].

At last, the field of so-called ‘therapeutic cancer vaccines’ has been growing in recent years. Earlier generations of vaccines had many pitfalls, such as, for example, insufficient antigen specificity and pronounced toxicity. Other challenges, including tumor heterogeneity and manufacturing complexity, have hampered their clinical translation [55]. However, advances in genomics and AI have enabled some of these challenges to be partially overcome, and personalized neoantigen vaccines are currently under technologically demanding development. Their efficacy and tolerability are still being monitored [56,57], as these medical products target the immune system and its capacity to recognize tumor-specific antigens [58,59,60]. Available clinical results have been observed in high-risk post-surgery patients, as demonstrated in the KEYNOTE-942 trial of individualized neoantigen mRNA-4157 (V940) plus pembrolizumab [61]. Molecularly targeted and immune-based therapies currently dominate pharmacological treatment protocols for RCC, but a growing interest in orthomolecular and natural product-based interventions targeting tumor metabolism, redox homeostasis, epigenetic regulation, and non-coding RNA networks exists. Many researchers and clinicians suggest that these options may be useful in disease management, in addition to potentially enhancing sensitivity to conventional therapies and mitigating resistance [62,63,64,65]. These modalities will be systematically analyzed in subsequent chapters regarding their mechanisms, preclinical evidence, and translational potential [66,67,68,69]. In the presented review, we accordingly present a synthesis of clinical, immunological, and molecular insights into RCC and discuss emerging biomarker-guided and metabolically oriented therapeutic strategies. The subsequent chapters will show, in more detail, relevant molecular aspects of RCC, such as B-cell signatures, KIM-1, tumor stemness, metabolic interventions, and translational challenges, providing a coherent framework linking multi-omics discoveries to personalized treatment approaches.

In summary, immune checkpoint inhibitors and VEGF/tyrosine kinase-targeted therapies remain the clinical backbone of advanced renal cell carcinoma management, and the present review deliberately places these established modalities within a broader integrative framework that also includes emerging and experimental approaches. Investigational strategies such as cellular therapies, therapeutic vaccines, and metabolic or orthomolecular interventions are discussed in greater mechanistic depth due to their rapidly expanding preclinical and translational literature. This therapeutic evolution underscores the importance of integrating tumor biology, immune context, and molecular stratification. The ideal goal would be to achieve a maximally optimized treatment selection approach.

### Bridging RCC Biology and Therapeutic Strategies

The molecular and cellular landscape of RCC is characterized by distinct biological processes researched in recent decades. These include disrupted hypoxia signaling pathways, metabolic reprogramming, and the development of a complex tumor immune microenvironment. Angiogenesis may, for example, be driven by loss of VHL function and subsequent stabilization of hypoxia-inducible factors (HIFs) in the context of altered cellular metabolism. Shifts in oxidative phosphorylation, glycolysis, and lipid metabolism also contribute to tumor growth and observed therapeutic resistance. It has to be particularly emphasized that RCC is characterized by variable infiltration of T cells, B cells, and myeloid populations in the tumor microenvironment, giving rise to distinct “immune hot” and “immune cold” phenotypes. These phenotypes accordingly respond differentially to treatments. These interdependent features underly the development of emerging therapeutic strategies. This is why RCC therapy aims to target both tumor-intrinsic pathways (e.g., angiogenesis and oncogenic signaling) and tumor–immune interactions.

In addition, biomarker-driven stratification has become a central concept in the clinical management of RCC. It provides a good framework that links tumor biology with therapeutic decision-making. In particular, RCC biomarkers derived from different omics profiling approaches are increasingly used gain information on hypoxia signaling and metabolic reprogramming in tumor cells, along with signaling and status in the tumor microenvironment. Such an integrative approach may aid in the identification of biologically distinct tumor subgroups, i.e., immune-inflamed and immune-excluded phenotypes. Indeed, omics-driven data gathering for stratification may allow for a very different choice of therapy, as RCC subtypes may differ substantially in their response to immune checkpoint blockade, targeted therapies, or even combination regimens. Clinical decision-making driven by omics biomarkers may improve the assessment of which patients are most likely to benefit from specific therapeutic modalities while sparing others from unnecessary toxicity. Omics biomarkers may accordingly serve as a translational bridge between molecular discovery and precision oncology in RCC [70].

Despite substantial therapeutic progress, metastatic renal cell carcinoma (mRCC) remains an area of significant unmet clinical need, and important limitations of current immune checkpoint inhibitor (ICI) and VEGF-targeted therapies persist in advanced renal cell carcinoma (RCC). Although combination regimens have improved efficacy and quality of life, overall response rates remain incomplete, and a significant proportion of patients exhibit primary resistance or fail to achieve durable long-term benefit. For example, long-term follow-up of pivotal trials such as CheckMate 214 and KEYNOTE-426 demonstrates that, while objective responses are observed in a subset of patients, sustained complete or durable responses remain limited, with many patients ultimately experiencing disease progression [39,71]. It is also important to note that these trials differ in design, patient stratification, prior treatment exposure, and endpoint definitions (e.g., PFS vs. OS vs. objective response rate), which may limit direct cross-trial comparability.

Furthermore, both intrinsic and acquired resistance mechanisms significantly constrain therapeutic efficacy. These include tumor-intrinsic factors such as impaired antigen presentation and interferon signaling, as well as tumor microenvironment-mediated immunosuppression driven by myeloid-derived suppressor cells, tumor-associated macrophages, and hypoxia-related pathways [72,73]. In parallel, resistance to VEGF-targeted therapies is associated with the activation of alternative angiogenic pathways (e.g., FGF, MET, and AXL) and metabolic adaptation, enabling tumors to bypass VEGF dependency [73]. Treatment-related toxicity also represents a major limitation, particularly in combination regimens, where grade 3 or 4 adverse events are frequent, necessitating dose modifications or treatment discontinuation in a subset of patients [74]. Importantly, predictive biomarkers remain insufficiently validated, and commonly used markers such as PD-L1 expression have shown limited utility in guiding treatment selection in RCC [71]. Finally, therapeutic options following progression on ICI-based combinations remain suboptimal, with limited evidence supporting effective salvage strategies, particularly after prior immunotherapy exposure [75]. These challenges are further compounded in non-clear-cell RCC, where clinical evidence is limited and treatment approaches are often extrapolated from clear-cell disease [76,77]. These limitations underscore the need for novel therapeutic strategies and improved treatment sequencing, providing a strong rationale for the development and earlier integration of emerging approaches such as cellular immunotherapies, personalized cancer vaccines, and myeloid-targeting strategies.

## 2. CAR-T Cells and Therapeutic Vaccines in Renal Cell Carcinoma: Current Evidence, Challenges, and Prospects

A significant proportion of patients with metastatic RCC progress in spite of currently available immunotherapy approaches. This is why next-generation immunotherapies have been continuously researched and the current scientific focus is particularly on cellular and so-called ‘vaccine-based’ strategies [33,34,39,40]. Some emerging approaches include chimeric antigen receptor-T (CAR-T) cells and therapeutic cancer vaccines designed to exert antigen-specific cytotoxicity and durable tumor-directed immune memory, respectively [78,79]. As previously elaborated, modulation of the tumor microenvironment can enhance CAR-T efficacy. Moreover, localized interferon signaling can enhance immune priming and antigen presentation, as supported by data from preclinical models in non-RCC systems. Accordingly, some authors suggest that these data provide a good rationale for exploring combinations with vaccines or checkpoint inhibitors rather than moving into research on the establishment of direct evidence of efficacy [80,81]. Therapeutic cancer vaccines exploit the idea of personalized neoantigens or tumor-associated antigens to expand polyclonal T-cell repertoires and improve immunological memory. Such strategy is seen as potentially synergistic with CAR-T strategies and checkpoint blockade. Integration with B-cell and T-cell multi-omics data, as we explain in detail later in the chapter on multi-omics and biomarkers, may help in the identification of biomarkers predictive of CAR-T cell persistence, antigen-specific expansion, and functional phenotypes.

### 2.1. CAR-T Cell Therapy for RCC

CAR-T therapy redirects T cells via synthetic receptors to recognize tumor-associated cell-surface antigens independent of HLA presentation. Translation to RCC depends on the identification of antigens that are sufficiently expressed on the malignant renal epithelium yet limited on essential normal tissues to minimize on-target/off-tumor toxicity. Candidate targets for RCC include CAIX, CD70, ROR1, MET, and B7-H3. These were chosen according to the available RCC expression profiling and early translational CAR-T targeting studies [82]. For example, early clinical experience with CAIX has shown that antigen expression on non-tumor tissues can provoke clinically significant toxicity. B7-H3 is also an attractive antigen in multiple solid tumors, including subsets of RCC. It is often overexpressed in tumors, with a relatively low distribution in normal-tissue. Deng et al. reported a second-generation CAR construct targeting B7-H3 where they were able to demonstrate antigen-dependent cytotoxicity against RCC cell lines and tumor growth inhibition in murine xenograft and orthotopic models [83]. These findings fit well in previous research on immune priming in multiple solid tumor models where functional activity and persistence of adoptively transferred T cells, including CAR-T platforms, were improved [84,85]. Nevertheless, this evidence comes mostly from interferon-based studies that were done outside of RCC and outside of CAR-T therapy contexts [80,81]. Clinical experience in RCC is still lacking in terms of the exploitability of the identified targets. The CAIX-directed CAR-T approach was demonstrated to be antigen-specific, but the dose-dependent hepatotoxicity attributable to CAIX expression on bile ducts hampers further translation [82]. Safety strategies to minimize systemic exposure are accordingly being evaluated and studied. Some of them include inducible safety switches, transient CAR expression, combinatorial antigen recognition (AND/NOT gates), and regional delivery. Modern genetic CAR-T constructs are designed to bear optimized co-stimulatory signaling domains and “protective” features such as cytokine expression or checkpoint-resistance modules to increase intratumor persistence and resistance to the suppressive tumor microenvironment [86,87,88,89].

Multi-omics profiling may be useful in the design of these constructs, as it may reveal patient-specific T-cell phenotypes and microenvironmental features relevant for assessing the potential fate of CAR-T trafficking and persistence. It is well known that many biological and technical hurdles impede CAR-T gene therapy, including antigen heterogeneity and on-target/off-tumor toxicity. As is the case for many other tumors, RCC exhibits intra- and inter-patient heterogeneity. CARs that recognize only one antigen may also fail under in vivo conditions, as tumor cells may lose that specific antigen. Limited tumor specificity can underlie substantial damage to healthy tissues. It is accordingly more rational to test CAR designs that require recognition of two antigens, as this may increase targeting accuracy. Moreover, an immunosuppressive tumor microenvironment (TME) with suppressive myeloid populations and hypoxic regions impairs T-cell infiltration and function. This is why therapeutic approaches include drugs that reduce immune suppression or alter tumor blood vessels, such as engineered CAR-T cells to produce supportive cytokines, all delivered locally for a short period [90,91]. Checking and profiling of the patient-specific immune status, i.e., by use of B-cell and T-cell multi-omics analyses, prediction of the T-cell exhaustion risk may be assessed in each patient. At last, cytokine release syndrome, neurotoxicity, and on-target effects cannot be ruled out. This is why monitoring, combined with anticipation of novel toxicities in solid tumors, is required. While current state-of-the-art methods are available for this purpose, the feasibility of their implementation with an individualized patient approach in the routine clinical environment remains questionable.

### 2.2. Therapeutic Vaccines for RCC

In recent years, efforts have been put into development for therapeutic vaccines for RCC that primarily aim to prime or expand tumor-specific T cells. Different vaccine platforms have been tested in RCC and other solid tumors, including peptide vaccines, dendritic cell (DC)-based vaccines, and viral vector platforms [56]. Earlier interferon- or monocyte-based delivery systems mainly provided mechanistic insights rather than being translated into direct clinical vaccine platform development [80,81]. Cell-based vaccines for renal cell carcinoma (RCC) that use irradiated autologous tumor cells or tumor antigens to stimulate a cytotoxic T-lymphocyte (CTL) response have been proposed, i.e., the vaccine Reniale^®^ (AGS-003), which is a dendritic cell-based vaccine that uses mature dendritic cells electroporated with tumor mRNA and CD40L to enhance co-stimulation, reverse T-cell anergy from PD-1, and promote T-cell activation. This vaccine has been tested in clinical trials, showing only some improvement in progression-free survival. Autologous vaccines are often delivered along with adjuvants such as BCG, IL-2, IL-12, and GM-CSF to enhance immune activation [92]. Better results have been obtained by neoantigen approaches. Such approaches are compelling in RCC because tumor sequencing frequently identifies clonal and subclonal mutations in driver genes such as VHL, PBRM1, and BAP1 [34,40]. Personalized neoantigen cancer vaccine trials in high-risk RCC have reported robust immunogenicity. For this purpose, neoantigens are derived from tumor-specific mutations and act as targets of T cell-mediated antitumor immunity. Vaccinated patients developed T-cell responses against vaccine peptides, with durable peripheral clonal expansion and ex vivo recognition of autologous tumors [79,93]. Challenges with respect to the use of personalized cancer vaccines as adjuvant therapy in tumors are mainly related to tumors with a low mutational burden. Recent advancements include studies involving the use of mRNA cancer vaccines targeting tumor antigens and neoantigens [94]. These mRNA vaccines are still considered and researched as an early therapeutic strategy in RCC immunotherapy. The basic technology of these products involves the use of messenger RNA encoding tumor-associated or neoantigenic sequences to produce tumor-specific T-cell responses. Some already identified potential targets in clear-cell RCC neoantigens are, for example, TOP2A, NCF4, FMNL1, and DOK3. Interestingly, in a recently published study, distinct immune subtypes were identified in clear-cell RCC with different prognoses, which points to the need for personalization of therapeutic strategies, as certain patient groups seem to have the potential to benefit more from mRNA vaccine therapy [95]. Trials studying the effects of currently available mRNA-based vaccines in combination with immunotherapies are already underway (Table 2) in investigational stages or phase I/II. For example, phase I/II early-stage clinical trials have been performed in China [96,97]. Russian scientists have also reported positive clinical mRNA clinical results and potential for further development in the treatment of cancer. Nevertheless, Russian efforts are from early translational or exploratory studies, as, at the moment, publicly verifiable phase results for RCC vaccines are still not available. In this field, many challenges related to in vivo mRNA platform behavior remain to be addressed, in addition to evaluation of therapeutic safety. 

At last, oncolytic virus vaccines (OVVs) and viral vector vaccines have also been tested. They were found to induce in situ inflammation and antigen release, potentially converting immunologically “cold” metastases poorly infiltrated by T-cells into “hot” lesions receptive to T-cell attack but also more susceptible to checkpoint blockade therapies such as, for example, PD 1/PD L1 inhibitors [37,111]. Some recent reports have provided information about ongoing clinical trials combining vaccinia OVVs with ICIs, though the results have still not been publicly disclosed. One RCC-focused clinical trial based on the study of an oncolytic vaccinia virus (JX-594) in combination with a PD-1 inhibitor suggests early-phase safety and efficacy assessment in early-stage mRCC model mice [112]. Accordingly, it may be plausible to test the use of vaccines in combination with checkpoint blockade or other agents that relieve immune suppression and enhance T-cell trafficking [37,80,81,90,91]. The current promise of cancer vaccines is mainly seen as an addition to existing therapeutic combinations. Such a combined approach in treatment should be tested due to its potential in reducing tumor burden and cancer-mediated immunosuppression in post-surgery settings. An overview of currently available RCC vaccines is presented in Table 3.

These findings support the rationale for further investigation of CAR-T therapy in combination with cancer vaccines for enhancement of immune response through expansion of the T-cell repertoire and epitope spreading. The boosting of CAR-T cells by cancer vaccines has, indeed, been shown to promote recruitment of antigen-presenting cells and activation of endogenous T cells. This allows for the recognition of antigens beyond the original CAR target, resulting in epitope spreading [121,122,123]. Therapeutic strategies that integrate CAR-T cell therapies with vaccine-based priming are supported by strong theoretical and preclinical rationale, although direct experimental evidence in RCC remains limited and is largely extrapolated from non-RCC solid tumor systems. In addition, we should emphasize that CAR-T cells directed against tumor-associated antigens may also recognize normal tissues. This has already been documented in tumor therapy as so called on-target/off-tumor toxicity, which may lead to organ damage, cytokine release syndrome, and immune-mediated adverse events—sometimes leading to fatal outcomes [121,124]. This is why such an approach requires more research and particularly careful antigen selection and monitoring for immune-related toxicities in combined CAR-T and vaccine therapies.

### 2.3. Integrative Perspective on CAR-T Cells, Therapeutic Vaccines, and Immune Checkpoint Inhibitors

CAR-T cell therapy, therapeutic cancer vaccines, and immune checkpoint inhibitors are often discussed as distinct immunotherapeutic modalities. However, they are mechanistically interconnected and share the immune activation cascade. In particular, the central event common to all three of these approaches includes processes of antigen availability, antigen presentation, and T-cell priming. Therapeutic vaccines primarily act at the level of antigen supply and dendritic cell-mediated priming. This leads to expansion of tumor-specific T-cell repertoires. Engineered CAR-T cell therapies bypass conventional antigen presentation, as effector T cells recognize tumor-associated antigens in an MHC-independent manner. However, it should be noted that their activity is influenced by endogenous antigen presentation and the inflammatory status of the tumor microenvironment.

Immune checkpoint inhibitors primarily modulate the inhibitory signaling pathways that regulate T-cell activation and exhaustion. In such a way, they may enhance the effect of vaccine-induced and CAR-T-mediated immune responses. The tumor microenvironment plays a crucial role in these therapeutic approaches, as factors such as hypoxia, immunosuppressive myeloid populations, and antigen heterogeneity underly the magnitude and durability of antitumor immunity. In this context, combination strategies are increasingly rationalized, as they simultaneously enhance antigen priming (vaccines), antigen recognition (CAR-T), and effector reinvigoration (checkpoint blockade).

In summary, immune targets and signaling pathways in RCC are not isolated entities but may be seen as components of an interconnected regulatory network. Understanding these interactions is essential for the rational design of combination immunotherapies in RCC. Moreover, there is growing interest in expanding the landscape of immunotherapeutic targets toward more context-specific regulators of immune signaling. This may be seen as a conceptual shift from single-pathway checkpoint inhibition toward a targeting of a broader immune network governing antigen presentation, T-cell activation, and myeloid-mediated immunosuppression. Future immunotherapeutic strategies are likely to be directed to dynamic immune signaling nodes rather than a limited set of canonical checkpoints. This is why integration of the emerging immune targets with multi-omics-based immune profiling may provide a rational basis for patient stratification.

## 3. TFE3 Fusion Proteins in Translocation Renal Cell Carcinoma

Translocation renal cell carcinoma (tRCC) is a rare but clinically significant and aggressive form of kidney cancer. It is commonly underlined by Xp11 TFE3 gene translocation. These chromosomal translocations cause fusion of TFE3, a member of the MiT/TFE transcription factor family, to a variety of partner genes. The results of these genetic rearrangements are chimeric proteins with aberrant transcriptional activity driving oncogenesis [125]. tRCC may present with aggressive clinical behavior, often occurring in young adults. Patients show variable responses to conventional RCC therapies, including VEGF-targeted agents and immune checkpoint inhibitors [33,34,126,127,128]. The therapeutic management of these patients is hampered by the fact that the molecular mechanisms of tRCC pathogenesis are still elusive. Mechanistic understanding and novel therapeutic strategies have been accordingly researched to overcome the limited efficacy of standard RCC therapies in this subtype. Recent studies have pointed to profound metabolic rewiring, lipophagic enhancement, and ferroptosis resistance programs in tRCC that may be evaluated as potentially novel therapeutic targets [126,127,128]. Herein, we examine the role of TFE3 translocations in tRCC, integrating molecular and clinical data to inform potential therapeutic strategies.

Multiple fusion partners have been identified for TFE3 in tRCC. These are ASPSCR1 (ASPL), PRCC, SFPQ, NONO, and CLTC, with ASPSCR1-TFE3, PRCC-TFE3, SFPQ-TFE3, and NONO-TFE3 being the most frequent [125,129]. These fusion proteins retain functional domains that are critical for DNA binding and dimerization, i.e., the basic helix–loop–helix (bHLH) and leucine-zipper regions [125,130]. ASPSCR1-TFE3 fusion cases were found to more likely present with regional lymph-node metastasis, followed by PRCC-TFE3 fusion cases. tRCC patients who presented with distant metastases and had poor outcomes had the ASPSCR1-TFE3 fusion type. PRCC-TFE3 fusion tumors had later recurrences and were less commonly node-positive [131,132]. In addition, NONO-TFE3 and SFPQ-TFE3 have a better survival outcome. Altogether, molecular subclassification is, indeed, of clinical relevance [125,133]. Emerging data suggest that the integration of molecular subclassification with clinical parameters and therapeutic response could improve patient stratification, particularly given the limited efficacy of VEGF-targeted therapies and ICIs in tRCC [33]. TFE3 immunohistochemistry is already widely used for diagnostic purposes in spite of certain technical variations. For example, FISH with break-apart probes yields a higher specificity but may miss cryptic intrachromosomal rearrangements. It seems that RNA sequencing is an adequate method for accurate molecular diagnosis, as it may detect both canonical and rare fusion partners [125,129,133]. TFE3-rearranged RCCs are morphologically heterogeneous. ASPL/ASPSCR1–TFE3 tumors often show a nested architecture; voluminous, clear cytoplasm; and psammoma bodies, whereas PRCC–TFE3 cases are more compact, with a less voluminous cytoplasm. SFPQ–TFE3 tumors can display pseudorosettes, small-cell clusters, or branching tubules. Immunohistochemistry typically shows strong nuclear TFE3, PAX8, and CD10 positivity, with negative staining for TFEB, HMB45, Melan-A, and Cathepsin K [125,133,134]. Mechanistic studies indicate that TFE3 fusion proteins, particularly NONO–TFE3 and SFPQ–TFE3, form nuclear biomolecular condensates via phase separation. These condensates act like a ‘hub’ for recruitment of transcriptional coactivators, and they consequently induce chromatin remodeling and activation of oncogenic transcriptional programs. Condensate formation relies on the coiled-coil domain of the fusion partner, and the disruption of these condensates may abrogate transcriptional activation of key target genes [135]. This finding may be exploited for the development of new therapies that are designed to target the condensate-forming properties of TFE3 fusions via small molecules, peptides, or engineered proteins.

In a recent paper by Li et al., it was shown that TFE3 fusions transcriptionally rewire tRCC toward oxidative phosphorylation (OXPHOS) [126]. This finding is in contrast with the prevalent glycolytic metabolism of most other renal cancers [136,137]. This metabolic switch towards oxidative phosphorylation is accompanied by heightened glutathione levels found in renal cancers, which make tRCC sensitive to reductive stress. CRISPR screens identified EGLN1 as a key regulator of this process, and its inhibition destabilizes HIF-1α, disrupts OXPHOS, and impairs tumor proliferation. The authors accordingly argued that this specific target may be further researched as a therapeutic option for metabolic rewiring of tRCC [126]. Lipid metabolism is also impaired in this type of cancer, and TFE3 fusions are found to enhance chaperone-mediated autophagy (CMA) of lipid droplets via upregulation of LAMP2A. This lypophagy is a relevant event for the promotion of mitochondrial fatty-acid oxidation, as it promotes oxidative phosphorylation and tumor growth [127]. Moreover, TFE3 fusions activate transcriptional programs conferring resistance to ferroptosis, which also supports oxidative phosphorylation during phenotypic transitions and facilitates tumor aggressiveness. Interactions with the tumor microenvironment, such as the enrichment of myofibroblastic cancer-associated fibroblasts, further enhance these properties [128]. Therapeutic implications from these recent metabolic insights may inform the study and development of new molecules and therapeutic strategies directed towards disruption of oxidative phosphorylation dependence through EGLN1 inhibition, the targeting of lipophagy by blocking of LAMP2A or CMA, ferroptosis sensitization, or various combination approaches based on stratification of patient populations according to the fusion partner.

### Metabolic Convergence Between TFE3-Rearranged RCC and Hypoxia-Driven Signaling

TFE3-rearranged RCCs have a distinct genetic origin, but they share convergent biological features with other RCC subtypes. This is particularly true for metabolic reprogramming processes and adaptation to cellular stress. Indeed, TFE3 fusion proteins act as aberrant transcriptional regulators of lysosomal biogenesis, autophagy, and metabolic gene expression. This is how nutrient utilization enhances cellular survival under RCC stress conditions. These processes overlap functionally with pathways driven by hypoxia signaling and metabolic rewiring in more common RCC subtypes. In particular, both TFE3-driven tumors and VHL-deficient clear-cell RCC have increased glycolysis, altered mitochondrial function, and enhanced survival in hypoxic tumor microenvironments. These shared features mechanistically bridge rare translocation-associated RCC and canonical hypoxia-driven RCC types. Accordingly, below, we provide a discussion of HIF-2α signaling that should be interpreted not only in the context of VHL loss but also as part of a broader RCC metabolic adaptation program that spans multiple molecular subtypes.

## 4. HIF-2α Inhibitors in Clear-Cell Renal Cell Carcinoma

In recent years, scientific evidence has pointed to a new therapeutic target: stabilization and accumulation of hypoxia-inducible factors such as HIF-2α. Indeed, ccRCC is characterized by inactivation of the von Hippel–Lindau (VHL) tumor-suppressor gene. Its loss of function leads to constitutive stabilization of HIF-2α. HIF-2α is well known for its role in angiogenesis, erythropoiesis, and metabolic reprogramming, often through induction of VEGFA, EPO, and SLC2A1 [120,138]. Accordingly, this key molecular driver in ccRCC may be further studied as a novel approach in therapy, especially knowing that VEGF-targeted therapies and immune checkpoint inhibitors still induce substantial resistance [139,140]. Recent studies clearly point to metabolic dysregulation in RCC contributing to HIF-2α-driven tumor progression. This is why novel combination strategies may be designed in such a way as to target both HIF-2α and metabolic vulnerabilities to increase efficacy [62,65]. One promising therapeutic agent that selectively disrupts HIF-2α transcriptional activity is belzutifan, the first approved transcription-factor inhibitor for solid tumors. Belzutifan is currently being tested in clinical trials in patients with VHL-deficient ccRCC [141] and represents a substantial shift in therapeutic design towards oncogenic transcription targets rather than downstream effectors [142,143]. In normal cells with normal oxygen levels, HIF-2α is hydroxylated by prolyl hydroxylases, recognized by VHL E3 ubiquitin ligase, and consequently degraded in the proteasome. As ccRCC is VHL-deficient, HIF-2α fates change, and it evades degradation. In particular, it dimerizes with HIF 1β (ARNT) and binds hypoxia response elements, which activates the transcription of pro-survival, angiogenic, and metabolic genes [120,138]. Belzutifan is a selective HIF-2α inhibitor; in particular, it prevents the dimerization of HIF-2α with HIF-1β (ARNT). As such, HIF-2α’s ability to activate downstream oncogenic genes is compromised. This is how it inhibits tumor growth and angiogenesis [140,142]. Preclinical studies in ccRCC cell lines and murine xenograft models show that belzutifan inhibition of HIF-2α reduces VEGF expression, impairs tumor vascularization, and slows proliferation [144,145]. Some papers also report enhanced efficacy and adaptive resistance management in combinations of HIF-2α inhibition with metabolic and redox-targeted adjuncts such as, for example, high-dose vitamin C and β-hydroxybutyrate. However, these results are only experimental and have not yet been confirmed in clinical trials [68,69]. Preclinical and translational studies also suggest that combination strategies targeting HIF-2α alongside VEGF pathways, immune checkpoint blockade, or metabolic modulators (e.g., β-hydroxybutyrate and vitamin C) may enhance antitumor efficacy [69,140,143,144]. Even though inhibition of HIF-2α is highly promising, on-target effects of HIF-2α inhibition have been reported that require additional studies and clinical monitoring. These include decreased erythropoietin production, potentially causing anemia, and modulation of oxygen-sensing pathways [146,147]. For example, belzutifan showed relevant antitumor activity in both sporadic and VHL-associated ccRCC. In the Phase III LITESPARK-005 trial, for example, belzutifan was compared with everolimus in previously treated advanced ccRCC, and it proved to improve progression-free survival and disease control [120,148]. Patients with VHL-associated ccRCC exhibited higher response rates, and the overall response rates ranged from 30 to 45%, with durable responses in non-resected lesions [142]. However, severe side effects were monitored in 15% of belzutifan-administered patients, including anemia, hypoxia, anaphylaxis reaction, retinal detachment, and central retinal vein occlusion [146,147,149,150]. However, most events were mild to moderate and manageable with dose adjustment or supportive care. Some authors suggest that because belzutifan lacks the cardiovascular and renal toxicities typically associated with VEGF-targeted therapies, it may be suitable for combination with other systemic agents [144]. Acquired resistance to HIF-2α inhibitors may occur due to secondary mutations in HIF-2α, activation of alternative angiogenic or metabolic pathways, or changes in the tumor microenvironment [144,145,151]. Authors have proposed that one potential strategy to overcome adaptive resistance is the integration of metabolic and oxidative stress modulators in the therapeutic regimen [62,68]. This is why next-generation inhibitors with improved selectivity and pharmacokinetics are sought, as suggested by Dogra & Vaishampayan [152].

## 5. Temferon as an Autologous HSPC-Based IFN-α Gene Therapy for Metastatic Renal Cell Carcinoma

Advanced metastatic RCC remains the main treatment challenge, as a substantial subset of patients do not achieve durable responses with application of immune checkpoint inhibitors and TKI VEGF-targeted therapies [153]. Innovative approaches are sought that would ideally be capable of reshaping the tumor microenvironment. Below, we present our analysis of Temferon’s mechanisms of action and highlight its potential impact on immune modulation in RCC. Temferon is an autologous hematopoietic stem/progenitor cell (HSPC) gene therapy platform engineered to deliver interferon-α2 (IFN-α2) selectively into tumor tissue via differentiation into Tie2-expressing monocytes/macrophages (TEMs). The objective is to achieve sustained intratumoral IFN-α activity while minimizing systemic exposure [90]. Preclinical studies have shown that TEM-mediated delivery of IFN-α is associated with transcriptional and functional shifts consistent with a transition toward a pro-inflammatory myeloid phenotype, enhanced antigen presentation, and increased intratumoral CD8^+^ T-cell infiltration, providing a mechanistic rationale for its use in metastatic RCC.

### 5.1. Mechanistic Rationale and Preclinical Evidence

Temferon has three experimentally demonstrated principles that underlie its potential in RCC treatment: (a) a subset of circulating myeloid cells characterized by Tie2 expression (TEMs) preferentially homes to tumor-associated vasculature and accumulates in the tumor microenvironment; (b) off-target IFN-α production is prevented by Tie2-dependent transcriptional regulatory elements that restrict transgene expression to TEM progeny; and (c) localized interferon type I within tumors exerts strong antitumor effects, along with reduced systemic toxicity in comparison with systemic cytokine therapy. For example, De Palma and colleagues have shown that in murine tumor models, Tie2-expressing monocytes engineered to deliver IFN-α home to tumor vasculature inhibit angiogenesis, reduce tumor growth, and activate innate and adaptive antitumor responses. Moreover, the observed biological effects are consistent with modulation of the tumor myeloid compartment, without impairing systemic myelopoiesis or wound healing. This important preclinical proof of concept for Temferon clearly shows a mechanistic link between TEM-restricted IFN-α delivery and TME reprogramming [90]. Further on, complementary preclinical studies including intratumoral IFN-α gene transfer and HSPC-mediated cytokine delivery showed that localized IFN-α enhances antigen presentation, increases T-cell recruitment via chemokine induction, and has been shown to enhance antitumor immune priming and is biologically consistent with potential combinatorial activity alongside immunotherapeutic strategies in preclinical models, supporting the biological plausibility of sustained intratumoral IFN-α activity as a means to counter tumor-associated myeloid immunosuppression [91]. These findings collectively support the concept of IFN-α-mediated myeloid repolarization as a therapeutic axis with potential relevance for RCC. Due to promising preclinical results, the Temferon platform entered clinical evaluation in a Phase I/IIa dose-escalation trial in newly diagnosed glioblastoma [80,81]. Available publications, including interim conference abstracts and company disclosures, indicate that autologous CD34^+^ HSPCs can be harvested, transduced with the lentiviral IFN-α construct, and reinfused after conditioning. Importantly, so far, the data shows that vector copy number, cell viability, and engraftment kinetics remained within expected ranges for lentiviral HSPC products. At last, no dose-limiting toxicities were observed in reported interim datasets [80]. Vector-marked myeloid progeny was found in peripheral blood and bone marrow, while gene-marked macrophages accompanied by transcriptional signatures consistent with type I interferon pathway activation and macrophage repolarization were measured in tumor specimens [81]. These results are encouraging but can still be assessed to be very preliminary and need further and scrutinous study and evaluation.

### 5.2. Rationale for Further Testing of Temferon in Management of Metastatic RCC

RCC tumors are known to often be enriched with myeloid cells, including tumor-associated macrophages, which promote immune suppression and resistance to ICIs and TKIs. The mechanisms that can be targeted in management of clear-cell RCC include myeloid-driven immunoregulation, angiogenesis, and hypoxia-induced transcriptional programs. Accordingly, therapies targeting tumor-resident myeloid populations may be very useful. In addition, type I interferon signaling remains biologically relevant in RCC, and prior systemic IFN-α therapy demonstrated modest but reproducible clinical activity. Temferon induces a local interferon-driven inflammatory program, enhanced antigen presentation, and augmented intratumoral CD8^+^ T-cell infiltration, as it restricts IFN-α production to tumor-infiltrating myeloid cells. This is a solid biological rationale for potential restoration or potentiation of responsiveness to ICIs or VEGF-targeted agents [153]. Preclinical results and mechanistic readouts from the TEM-GBM clinical trial further support this translational rationale. Moreover, recent network meta-analyses provide updated evidence for first-line ICI-based combinations in mRCC. Wang et al. (2025) reported a Bayesian network analysis of 30 RCTs (14,959 patients), showing overall the survival benefit of nivolumab + cabozantinib and pembrolizumab + lenvatinib, supporting the selection of combinatorial strategies in future RCC studies [154].

The Italian Medicines Agency (AIFA) approved the initiation of a Phase 1/2 clinical study of Temferon in metastatic clear-cell RCC by Genenta [155,156]. The study design accessible in a publicly available registry and company data: (a) single-dose Temferon infusion following conditioning. (b) Cohort stratification by prior ICI exposure (ICI-naïve cohort receiving pembrolizumab post Temferon; ICI-exposed cohort receiving cabozantinib upon progression). (c) Primary endpoints: safety and tolerability; (d) exploratory endpoints: persistence of gene-marked cells, intratumoral and peripheral biomarkers of IFN-α activity, and preliminary antitumor signals. Although the conditioning regimen is not specified in detail, it was probably aligned with myeloablative or reduced-intensity protocols that are typically used in HSPC-based lentiviral gene therapies. The eventual approval of Temferon is still pending.

### 5.3. Translational Rationale, Myeloid Reprogramming, and Biomarker Strategy

From a mechanistic point of view, immune evasion, as well as observed therapeutic resistance to ICIs and VEGF-targeted therapies, may be explained by the immunosuppressive tumor microenvironment (TME) in metastatic RCC, which is thoroughly infiltrated by myeloid cells, including tumor-associated macrophages (TAMs) and myeloid-derived suppressor cells (MDSCs) [42,78,153]. Accordingly, type I interferon (IFN α) signaling may be exploited in RCC therapy, as this signaling pathway enhances antigen presentation and induces pro-inflammatory chemokine secretion (e.g., CXCL9/10). It has also been established that it stimulates CD8^+^ T-cell recruitment and can repolarize myeloid cells from an immunosuppressive (M2-like) to an immunostimulatory (M1-like) phenotype [90,91]. Temferon fits well into this mechanistic context, as it exploits these principles. Temferon accordingly delivers IFN α locally through Tie2-expressing monocyte progeny (TEMs), and a reprogramming of the tumor myeloid compartment occurs. Importantly, it has been shown that in preclinical murine models, TEM-mediated IFN α delivery inhibits angiogenesis, reduces tumor growth, and potentiates both innate and adaptive antitumor responses without impairing systemic hematopoiesis or wound healing [90]. Intratumoral IFN α also has a synergistic effect with immunotherapeutic interventions. IFN α has, indeed, been associated with immune-activating microenvironmental changes in multiple preclinical systems. All these data support further testing of Temferon in combination with ICIs or VEGF-targeted agents [39,42,91]. While the explained rationale is well supported by preclinical data, it should be noted that translational biomarker strategies are critical for evaluating mechanistic proof and predicting response. Some of the proposed biomarkers were already mentioned in the literature, including the monitoring of vector-marked TEM progeny in blood and tumor tissue, profiling of interferon-stimulated gene (ISG) signatures in tumor biopsies, assessment of macrophage polarization states, and evaluation of T-cell infiltration and activation markers [78,80,81]. It is expected that these biomarkers may inform patient selection and combination strategies as well, particularly in high-risk RCC patients with myeloid-enriched TMEs. In addition, emerging evidence indicates that modulation of the myeloid compartment via localized IFN α may overcome adaptive immunosuppression and enhance sensitivity to ICIs and TKIs, a mechanism supported by clinical observations in RCC and preclinical synergy models [39,42]. Together, these data provide a scientifically rigorous rationale for Temferon’s therapeutic strategy in mRCC, integrating mechanistic myeloid reprogramming, biomarker-guided monitoring, and combinatorial synergy with existing immunotherapies.

### 5.4. Conclusions and Outlook

Temferon is a mechanistically distinct autologous HSPC-based gene therapy designed to re-educate the tumor myeloid compartment through sustained, localized IFN-α delivery. Preclinical data demonstrating tumor-targeted IFN-α activity [90], together with preliminary mechanistic findings from TEM-GBM [73,74], provide biological and translational justification for clinical evaluation in metastatic RCC rather than definitive evidence of clinical efficacy. Given the favorable outcomes from recent ICI + TKI combinations in first-line mRCC [157,158,159], integrating Temferon into sequential or combination regimens could be scientifically justified. The ongoing Phase 1/2 study [156] will be critical in assessing safety, engraftment durability, intratumoral biological activity, and early efficacy signals. If results are favorable, subsequent development would be translationally relevant and may offer a new paradigm for myeloid-targeted therapy in mRCC.

A critical general consideration for the clinical translation of advanced therapeutic modalities such as autologous hematopoietic stem/progenitor cell (HSPC)-based gene therapies (e.g., Temferon), CAR-T cell therapies, and personalized cancer vaccines is their manufacturing complexity and associated turnaround time. These approaches require patient-specific cell collection, genetic modification or antigen design, quality control, and reinfusion, which may take several weeks. During this period, patients with aggressive or progressive metastatic renal cell carcinoma (mRCC) may require bridging strategies to maintain disease control. In current clinical practice, such strategies could include continuation or initiation of VEGF-targeted tyrosine kinase inhibitors (TKIs); the use of immune checkpoint inhibitors (ICIs) as monotherapy or in combination; or ICI–TKI combinations, which remain the standard first-line treatment. These approaches may stabilize disease and preserve patient eligibility for subsequent advanced therapies. The concept of bridging is particularly relevant for patients with high tumor burden or rapidly progressive disease, where delays in treatment initiation could negatively impact outcomes. Based on current biological rationale and early clinical data, several potential integration points can be proposed, such as a post first-line setting, combination strategies, and a minimal residual disease/adjuvant setting, as shown in Table 4.

## 6. Orthomolecular and Natural Product Supportive Therapy in Renal Cell Carcinoma

In recent decades advancement have been seen in surgery, radiotherapy, and systemic treatments of cancer. Nevertheless, many solid tumors have high recurrence rates, and patients develop treatment resistance and suffer from significant toxicity. Some current oncological treatments may have limited long-term effectiveness in specific patient subsets due to resistance and toxicity. There is growing interest in more comprehensive and individualized approaches taking into account metabolic and nutritional factors underlying malignant diseases. Indeed, a considerable amount of published research points to metabolic changes and nutritional deficiencies as key players in cancer development. However, scientific publications largely discuss the application of synthetic molecules or drugs that target these core biological pathways [160] or propose dietary interventions that tackle the problem of nutrient availability, and metabolic dysfunction intersects with signaling pathways in tumorigenesis [161]. In recent decades, the orthomolecular approach has gained importance, as it is directed toward the correction of biochemical imbalances by application of vitamins, minerals, amino acids, fatty acids, antioxidants, and other endogenous or dietary bioactive compounds. The role of the orthomolecular approach in cancer therapy remains exploratory and requires rigorous validation, and further well-designed trials are required to determine its efficacy and safety. At present, the role of orthomolecular approaches in cancer therapy, including RCC, remains exploratory and hypothesis-generating. Their inclusion would, indeed, require rigorous clinical validation before conclusions can be drawn regarding therapeutic efficacy or integration into standard care.

Some approaches have already been explored in preclinical and translational models for their effects on redox balance, metabolic signaling, epigenetic regulation, and non-coding RNA networks [63,65]. These interventions affect pathways that have also been identified in multi-omics studies, suggesting potential combinatorial applications with immunotherapy and targeted therapies (based primarily on preclinical evidence). As current translational studies suggest uneven outcomes for immune checkpoint and targeted therapy efficacy in RCC, combinatorial approaches that include natural compounds have been evaluated in the context of the current lack of robust clinical confirmation [39,43,45,48,51]. Most evidence presented in this section is derived from in vitro and in vivo studies, and clinical validation in RCC patients remains limited. In this chapter, we present orthomolecular and natural product-based protocols, with emphasis on the mechanisms of action, preclinical evidence, and translational potential aligned with multi-omics findings (preclinical and mechanistic level) [66,67,68,69]. It has to be emphasized that natural compounds cannot be patented and therefore have not been a focus of the pharma industry so far.

### 6.1. Polyphenols and Flavonoids in RCC

Flavonoids, probably the most popular group of natural polyphenolic compounds, are found in fruits and vegetables, as well as in their derived products. Several flavonoids, such as, for example, apigenin, luteolin, quercetin, and (-)-epigallocatechin-3-gallate (EGCG), have shown anticancer activity in preclinical RCC models. For example, anticancer potential of apigenin, a strong antioxidant and anti-inflammatory flavonoid, has already been proven, mainly through its direct effect on tumor suppressor genes, angiogenesis, apoptosis, the cell cycle, inflammation, apoptosis, PI3K/AKT, NF-κB, MAPK/ERK, and STAT3 pathways [162]. It also inhibits proliferation in human RCC cell lines in vitro (ACHN, 786-O, and Caki-1), where it induces G2/M cell-cycle arrest, DNA damage, ATM signaling activation, and p53-dependent apoptosis. In mouse xenograft models, apigenin reduced tumor volume and the Ki-67 proliferation index without toxicity [163]. Similarly, EGCG, the most biologically active catechin polyphenol in green tea, with strong antioxidative, anti-inflammatory, and anticancer effects [164], has also been found to suppress invasion and migration of RCC cells in vitro through the mechanism of MMP-2 and MMP-9 downregulation. It also activates TFEB-mediated autophagy and inhibits epithelial–mesenchymal transition. The latter is highly relevant, as it suggests activity on metabolic and redox pathways identified in multi-omics studies [165,166]. Luteolin is a flavone flavonoid with broad biological activities including anticancer effects through its direct antiproliferative effect on cancer cells, apoptosis induction, angiogenesis, invasion, and metastasis inhibition, as well as modulation of the mTOR/PI3K/Akt, STAT3, and Wnt/β-catenin pathways [167]. Specifically, it inhibits PI3K/AKT and MAPK signaling, reduces proliferation, and induces ROS-mediated apoptosis in RCC cell models [62]. At last, quercetin, a flavonol with diverse proven biological activities [168], including anticancer effects, has been found to modulate PI3K/AKT signaling and oxidative stress, potentially enhancing chemotherapy or TKI sensitivity and acting on metabolic pathways relevant to B-cell-associated immune subtypes in RCC [63].

Flavonoids, in general, influence epigenetic modifications that are strongly disrupted in cancer cells, including DNA methylation and histone acetylation. They also regulate lncRNAs and miRNAs, which are crucial for tumor cell proliferation, metastasis, and drug resistance [63]. This impact is in line with the immune–metabolic heterogeneity observed in renal cell carcinoma (RCC) and uncovered in detail through recent multi-omics profiling studies that identified distinct metabolic and immune subtypes associated with tumor progression and clinical outcomes in ccRCC [169]. A general overview of the epigenetic effects of flavonoids is given in Table 5.

### 6.2. Other Naturally Derived Compounds with Potential in RCC Management

Allicin is a sulfur-containing natural product derived from garlic with strong biological activities. In RCC cell lines, it has been found to reduce HIF-1α, VEGF, and Bcl-2 expression, with a concomitant promotion of caspase-dependent apoptosis and cell migration. These effects correlate with hypoxia and metabolic pathways relevant to RCC subtypes, as described in different RCC multi-omics studies [182].

*Ginsenoside Compound K (CK)* has also been studied as a major intestinal metabolite of protopanaxadiol-type ginsenosides derived from *Panax ginseng.* It is produced by the gut microbiota and is the most biologically active and bioavailable ginseng metabolite. In the context of RCC, it has been shown to inhibit proliferation, migration, and invasion of RCC cell lines. It also induces cell-cycle arrest and apoptosis, increases intracellular ROS, and downregulates the THOR lncRNA. Again, the correlation of these effects may be corroborated with oxidative stress and epigenetic pathways revealed in RCC by multi-omics studies [64]. Moreover, the bioactive protopanaxadiol-type saponin derived from the same plant, ginsenoside Rg3, is one of the most extensively studied ginsenosides for anticancer activity. It is enriched by heat processing. It has been shown to reduce migration, invasion, colony formation, and tube formation, with a concomitant promotion of apoptosis. On a molecular level, it induces DNA demethylation and histone acetylation, which makes it an attractive compound for the development of combined strategies with TKIs or immunotherapy [183]. Ginsenoside Rh4 from heat-processed *Panax ginseng* is a minor protopanaxadiol-type saponin that is gaining researchers’ interest due to its anticancer, anti-metastatic, and immunomodulatory activities [184]. In the context of RCC, it has been found to increase RCC cell sensitivity to ferroptosis through suppression of NRF2 signaling and downregulation of GPX4, SOD1, and catalase. Again, the targeted mechanisms align with redox and metabolic pathways identified in B-cell and stemness-related RCC subtypes and provide a preclinical rationale for further investigation of potential combinatorial strategies [185].

Finally, vitamin C (ascorbate) has also been extensively investigated in cancer treatment, as has its rationale for applications in RCC. In preclinical studies, high-dose pharmacological ascorbate demonstrated selective cytotoxicity. It reduced HIF-1α stabilization; downregulated GLUT1, VEGF, and PDK1; disrupted glycolysis; increased mitochondrial ROS; and induced apoptosis. Again, the effects are aligned with main disrupted pathways in RCC identified by multi-omics approaches [68,186,187]. It should be noted that epidemiological studies show no strong protective effect of dietary vitamin C against RCC. Higher intake, however, may have a modest inverse association. In addition, high-dose intravenous administration carries a risk for renal injury in patients with impaired kidney function, which calls for controlled application and clinical evaluation of the supportive therapy [188]. RCC-specific clinical trials with vitamin C are still limited. For example, a phase II study of intravenous ascorbate with pazopanib was registered but terminated, highlighting the need for early-phase clinical evaluation [189,190].

One nanomedicine approach relying on carbon nanodots is worth mentioning as well due to the often poor bioavailability of natural compounds from the intestine. For example, herbal extract-derived carbon nanodots improved the delivery of phytochemicals in a study by Tian et al. [66]. The herbal extract precursor used to make the carbon nanodots in this study was ginsenoside Re from *Panax ginseng*. They found that the nanodots increased ROS, inhibited PI3K/AKT signaling, and suppressed tumor growth in RCC A498 cells and suppressed tumor growth in xenograft models. This strategy seems to be appropriate to address the bioavailability limitations [66].

In summary, early-phase trials of CK, Rg3, Rh4, or high-dose vitamin C should be pursued to assess pharmacokinetics, tolerability, and maximum tolerated dose in RCC patients. In addition, formulation innovations are urgently needed to tackle the issues of bioavailability. Some potential solutions can be found in nanocarriers, as reviewed in detail by Pavelic et al. [191]. More combination strategies integrating natural compounds with TKIs, immunotherapy, or anti-angiogenics should be explored, as the benefits for patients may be high. Safety and interaction studies are essential to evaluate potential adverse effects of standard therapies.

### 6.3. Metabolic and Dietary Interventions in RCC

RCC is not only a genetic but also a metabolic disease. This is suggested by previous research that explains the extensive metabolic reprogramming in glucose and lipid metabolism, glutamine dependence, and other nutrient pathways that support tumor growth, survival under hypoxic conditions, and immunosuppression within the TME. In particular, mutations in VHL and disrupted PI3K/Akt/mTOR and HIF pathways underly metabolic changes in RCC that are also correlated with mechanisms of resistance to conventional therapies [192]. In addition, dietary factors and nutrition have been found to influence RCC risk, progression, and patient outcomes, particularly the increased intake of vitamin C and vegetables [193]. Accordingly, strategies such as the adoption of a Mediterranean or ketogenic diet seem to provide good support for RCC treatment and corresponding therapy responses. The Mediterranean diet has been assessed to have some benefit for RCC prevention, but such a diet should also be compared in terms of antineoplastic advantage in the future relative to other types of diets high in antioxidants that may also serve the same purpose [194]. Ketogenic diets are also studied in RCC, as they shift energy metabolism toward ketone body utilization. This shift may reduce proliferation and tumor growth and have been proven to enhance response to immunotherapy in RCC models. Richard et al. reported that a ketogenic diet enhanced PD-L1 blockade efficacy and increased CD8^+^ T-cell infiltration and cytotoxicity, which led to decreased tumor growth [195]. In this context, an important primary ketone during ketosis can be mentioned to underlie the mechanistic rationale for a ketogenic diet: β-Hydroxybutyrate. It has been found to reduce hypoxia-induced angiogenesis, downregulate HIF-2α, and suppress multidrug resistance transporters. It has also been found to have synergistic effects with quercetin in terms of impairing vascularization and metabolic adaptation [69].

## 7. Conclusions and Future Directions for RCC Management

Scientific and clinical knowledge point to RCC as a biologically diverse and therapeutically challenging malignancy that is driven by genetic alterations, metabolic reprogramming, and immune evasion. Moreover, the need for subtype-specific therapeutic strategies is rapidly being acknowledged, particularly due described genomic fusion events such as TFE3 and TFEB rearrangements in rare translocation RCC subtypes that underly distinct molecular and metabolic features in these types of cancer. In recent years, clinical outcomes for advanced RCC have significantly improved due to the usage of targeted therapies and immune checkpoint inhibitors. Nevertheless, many patients develop therapeutic resistance or experience substantial adverse events. Such adverse events may be serious and include fatigue, anemia, hypertension, and organ-specific toxicities, i.e., linked to VEGF or HIF-2α inhibition. Accordingly, a first-in-class HIF-2α inhibitor, belzutifan, shows clinical efficacy in clear-cell RCC and VHL-associated disease, with concomitant toxicity and metabolic side effects. As growing evidence points to metabolic reprogramming as a central feature of RCC pathogenesis, other therapeutic options may be considered for this group of patients that act more interactively or in combination with existing therapeutics. In particular, alterations in glucose, lipid, and amino-acid metabolism offer attractive novel therapeutic targets for emerging metabolic inhibitors and dietary modulation strategies, e.g., targeting of glutamine or lipid pathways. In this area, available clinical data in RCC patients remains limited. At last, natural product compounds such as, for example, flavonoids may be used as supportive adjuncts to conventional therapy for modulation of metabolic signaling and oxidative stress. An expanded and integrated view of RCC pathogenesis underpins the value of combined metabolic and dietary interventions alongside precision-targeted agents and immunotherapies in RCC treatment. In such a way, treatment-related toxicity and enhanced quality of life may be achieved, along with improved therapy outcomes. Future research should accordingly focus on validation of personalized metabolic biomarkers, optimization of combination regimens, and studies on the usefulness of orthomolecular and dietary strategies in personalized care pathways for RCC.

## Figures and Tables

**Table 1:** Classification of RCC subtypes with corresponding molecular characteristics.

RCC Subtype	Target	Molecular Alteration Type	Biological Role	Role in Tumor/Metastatic Potential	Level of Evidence/Clinical Relevance	References
Clear-cell RCC	VHL	Tumor suppressor gene mutation/inactivation	Oxygen sensing via HIF regulation	Loss → HIF stabilization → angiogenesis, proliferation; high metastatic potential (lung, bone, and liver)	Strong clinical + translational evidence	[2,3,4]
Multilocular cystic RCC (low malignant potential)	VHL pathway	VHL pathway alteration	Same as above	Indolent; minimal to no metastatic potential	Well-established pathological entity	[5,6]
Papillary adenoma	Trisomy 7/17, MET	Chromosomal gains/proto-oncogene activation	Growth signaling	Small, benign lesion; no metastasis	Histologically defined benign lesion	[5,7]
Papillary RCC (pRCC)	MET	Receptor tyrosine kinase activation	Proliferation and survival signaling	Variable metastatic risk depending on subtype	Established molecular subclassification	[8,9,10]
Oncocytoma	Mitochondrial genome alterations	Mitochondrial DNA alterations	Energy metabolism	Benign; no metastasis	Well-characterized benign tumor	[5,11]
Chromophobe RCC	Multiple monosomies	Chromosomal losses	Tumor suppressor loss/mitochondrial dysregulation	Typically indolent; rare metastasis	Established entity	[5,8,12]
Low-grade oncocytic tumors (LOT, EVT, and HOCT)	Mitochondrial pathways	Mitochondrial alterations (heterogeneous)	Energy metabolism	Indolent; very low metastatic potential	Emerging entity/evolving classification	[5,11,13]
Collecting duct carcinoma	High-grade molecular alterations	Poorly defined/complex genomic alterations	Multiple oncogenic pathways	Highly aggressive; early metastasis common	Rare but clinically recognized aggressive RCC	[5,14]
Clear-cell papillary RCC	No VHL/trisomy 7/17	Distinct molecular profile (non-VHL-driven)	Oxygen sensing/epithelial signaling	Indolent; negligible metastasis	Recently defined entity with strong pathological consensus	[5,15]
Mucinous tubular and spindle cell carcinoma (MTSCC)	Hippo/YAP1 pathway	Pathway-level dysregulation	Cell proliferation and organ size regulation	Low-grade; rare metastasis	Rare but recognized subtype	[5,16,17]
Tubulocystic RCC	Trisomy 7/17 overlap	Copy-number alterations	Growth signaling	Mostly indolent; rare metastasis	Emerging entity with defined morphology	[5,18]
Acquired cystic disease-associated RCC (ACD-RCC)	KMT2C, TSC2	Epigenetic + mTOR pathway genes	Epigenetic regulation/growth signaling	Intermediate metastatic risk; CKD-associated	Emerging entity with growing clinical evidence	[5,19,20,21]
Eosinophilic solid and cystic RCC (ESC RCC)	TSC1/TSC2	mTOR pathway alterations	Growth regulation via mTOR signaling	Low-grade tumor; low metastatic risk	Emerging but increasingly recognized	[22,23]
RCC and NOS	Heterogeneous	Not specified	Variable	Variable behavior; metastasis possible	Diagnostic category (not molecularly defined)	[5,11]
TFE3-rearranged RCC	TFE3 gene fusions	Translocation/gene fusion	Lysosomal biogenesis and metabolic regulation	Often aggressive; high metastatic potential	Established MiT family translocation RCC	[5,24]
TFEB-altered RCC	TFEB rearrangements/amplifications	Gene rearrangement/amplification	Lysosomal biogenesis	Variable; often aggressive when amplified	Rare but well-defined molecular entity	[5,25,26]
ELOC-mutated RCC	ELOC (TCEB1) mutation	Component of VHL E3 ligase complex	Hypoxia signaling regulation	Rare; variable metastatic potential	Emerging molecular subtype	[25,27,28]
FH-deficient RCC	FH	Krebs cycle enzyme mutation	TCA cycle/metabolic regulation	Highly aggressive; early metastasis	Strong hereditary/metabolic cancer syndrome link	[5,25]
SDH-deficient RCC	SDH complex genes	Mitochondrial enzyme complex mutation	Respiratory chain function	Rare; low to moderate metastatic potential	Rare hereditary tumor syndrome	[5,25,29]
ALK-rearranged RCC	ALK fusion genes	Tyrosine kinase fusion	Growth signaling	Rare; variable metastatic potential	Rare but targetable alteration	[25,30,31,32]
SMARCB1-deficient renal medullary carcinoma	SMARCB1 loss	Chromatin remodeling defect	Epigenetic regulation	Very aggressive; early metastasis	Well-defined, highly aggressive entity	[5,11,25]

Abbreviations: RCC = Renal Cell Carcinoma; VHL = Von Hippel–Lindau; HIF = Hypoxia-Inducible Factor; MET = MET proto-oncogene, receptor tyrosine kinase; pRCC = Papillary Renal Cell Carcinoma; LOT = Low-grade Oncocytic Tumor; EVT = Eosinophilic Vacuolated Tumor; HOCT = Hybrid Oncocytic/Chromophobe Tumor; MTSCC = Mucinous Tubular and Spindle Cell Carcinoma; YAP1 = Yes-Associated Protein 1; ACD-RCC = Acquired Cystic Disease-associated RCC; KMT2C = Lysine Methyltransferase 2C; TSC2 = Tuberous Sclerosis Complex 2; mTOR = mechanistic Target of Rapamycin; CKD = Chronic Kidney Disease; ESC RCC = Eosinophilic Solid and Cystic RCC; TSC1 = Tuberous Sclerosis Complex 1; NOS = Not Otherwise Specified; ELOC = Elongin C; TCEB1 = Transcription elongation factor B subunit 1; FH = Fumarate Hydratase; SDH = Succinate Dehydrogenase; ALK = Anaplastic Lymphoma Kinase; SMARCB1 = SWI/SNF-related, matrix-associated, actin-dependent regulator of chromatin subfamily B member 1.

**Table 2:** Antigen targets for CAR-T and vaccine-based immunotherapy in renal cell carcinoma (RCC).

Antigen Target	Type	Biological Considerations	References
Carbonic Anhydrase IX (CAIX)	Tumor-associated surface antigen	Highly expressed in >90% of clear-cell RCC; target of CAR-T and vaccine approaches; on-target/off-tumor toxicity was observed in early CAR-T studies—liver toxicity in RCC patients treated with anti-CAIX CAR-T and cross-reactivity of T cells engineered against MAGE-A3 that caused cardiac toxicity.	[98,99,100]
CD70	Surface antigen for CAR-T	Strongly expressed on RCC; CD70 CAR-T products are under active clinical evaluation; observed off-tumor toxicity is low.	[98]
Tyrosine protein kinase Met (c-Met)	Receptor tyrosine kinase	Targeted in CAR-T approaches; expressed in subsets of RCC; clinical study did not show an objective response to treatment.	[101,102]
AXL receptor tyrosine kinase (AXL), Receptor tyrosine kinase-like orphan receptor 2 (ROR2)	Aberrant tyrosine kinase receptors	Overexpressed in RCC and not common in normal adult tissues, representing attractive CAR-T targets; undergoing clinical study in adult subjects with relapsed and refractory stage IV metastatic RCC.	[103]
DNAJB8 (heat shock protein Hsp40 chaperone family, subfamily B, member 8 protein) and HLA-peptide complexes	Cancer/testis or stem cell-associated antigen	Emerging target from preclinical data; restricted tissue expression and lower off-tumor risk; peptide–HLA complexes can be targeted to increase specificity.	[104]
Neoantigens (VHL, PBRM1, BAP1, KDM5C, PIK3CA)	Personalized tumor mutation-derived targets	Personalized cancer vaccine peptide that contains a neoantigen derived from a cancer driver mutation, including common RCC gene mutations in VHL, PBRM1, BAP1, KDM5C, and PIK3CA, as well as other pan-cancer driver mutations; neoantigen targeting stimulates durable T-cell responses; Phase I trial in high-risk, fully resected clear-cell RCC patients.	[79,105]
5T4	Oncofetal/tumor-associated antigen	Highly expressed in RCC and several solid tumors; vaccine MVA-5T4 elicited immune responses with limited toxicities and did not significantly improve overall survival.	[106,107]
HERV-E-derived peptides (CT-RCC-1)	Endogenous retrovirus antigen	Tumor-specific antigen expressed in most clear-cell RCC and absent in normal tissues; recruits T-cell responses without expected off-tumor effects and partial response and stable disease in a subset of patients.	[108,109,110]

Abbreviations: CAR-T = Chimeric Antigen Receptor T Cell; MAGE-A3 = Melanoma-Associated Antigen 3; CD70 = Cluster of Differentiation 70; c-MET = MET Proto-Oncogene, Receptor Tyrosine Kinase; DNAJB8 = DnaJ Heat Shock Protein Family (Hsp40) Member B8; Hsp40 = Heat Shock Protein 40; HLA = Human Leukocyte Antigen; VHL = Von Hippel-Lindau; PBRM1 = Polybromo 1; BAP1 = BRCA1-Associated Protein 1; KDM5C = Lysine Demethylase 5C; PIK3CA = Phosphatidylinositol-4,5-Bisphosphate 3-Kinase Catalytic Subunit Alpha; 5T4 = Trophoblast Glycoprotein; MVA-5T4 = Modified Vaccinia Virus Ankara Encoding 5T4; HERV-E = Human Endogenous Retrovirus Type E; CT-RCC-1 = Clear Cell Renal Cell Carcinoma-Associated Transcript 1.

**Table 3:** Summary table with details on therapeutic vaccine strategies tested in renal cell carcinoma (RCC).

Type of Vaccine	Description	Target/Mechanism	Clinical Status	References
AGS-003 (Reniale)	Autologous tumor cell-based	Irradiated tumor lysate → cytotoxic T lymphocyte response	Phase III adjuvant; improved progression-free survival but inconsistent overall survival	[113,114,115]
IMA901	Multipeptide cancer vaccine	Multiple tumor-associated peptides with GM-CSF injected after a single low dose of cyclophosphamide	Phase III (IMPRINT) did *not* improve overal survival when added to sunitinib	[116,117]
TG-4010	Viral vector (Modified Vaccinia Ankara-MVA)	Recombinant MVA expressing MUC1	Phase II in metastatic RCC; no objective clinical response was observed in evaluation of TG4010 efficacy and tolerability, either alone or in combination with cytokines in stable disease	[118]
MVA-5T4/TroVax	Viral vector (Modified Vaccinia Ankara MVA)	5T4 tumor-associated antigen in MVA vector	Tested in phase II/III mRCC; did not demonstrate overal survival benefit	[106]
Neoantigen vaccine (NeoVax)	Personalized neoantigen peptide/personalized cancer vaccine (PCV)	Mutations (driver-gene neoantigens) and patient-specific immune targeting	Early Phase I RCC trial: strong immunogenicity against multiple epitopes and polyfunctional memory T-cell responses	[79,105]
mRNA vaccine V940 (mRNA-4157)	Personalized mRNA	mRNA encoding patient’s tumor antigens	Phase II adjuvant with pembrolizumab in RCC	[119,120]
Oncolytic viral strategies	Replication-defective viruses targeting tumors	Oncolysis + immune activation	Preclinical/early work demonstrated immune synergy in models	[112]

Abbreviations: AGS-003 = Autologous Dendritic Cell–Based Renal Cell Carcinoma Vaccine (Reniale); GM-CSF = Granulocyte-Macrophage Colony-Stimulating Factor; TG4010 = Recombinant Modified Vaccinia Ankara Virus Expressing MUC1 and Interleukin-2; MVA = Modified Vaccinia Ankara; MUC1 = Mucin 1; 5T4 = Trophoblast Glycoprotein; mRCC = Metastatic Renal Cell Carcinoma; mRNA = Messenger RNA; V940 (mRNA-4157) = Personalized mRNA-Based Neoantigen Cancer Vaccine.

**Table 4:** Integration points of autologous hematopoietic stem/progenitor cell (HSPC)-based gene therapies (e.g., Temferon), CAR-T cell therapies, and personalized cancer vaccines.

Setting/Strategy	Suggested Integration Points
Post first-line setting	After progression on ICI–TKI combinations, therapies such as Temferon or CAR-T cells may be explored as second- or third-line approaches, particularly in patients with immunosuppressive tumor microenvironments.
Combination strategies	Temferon-mediated myeloid reprogramming may enhance responsiveness to ICIs, supporting investigation in combination or sequential regimens.
Minimal residual disease/adjuvant setting	Personalized cancer vaccines may be particularly suited for post-surgical or low-burden disease, where induction of durable immune memory is most effective.

**Table 5:** Epigenetic mechanisms of flavonoids in cancer treatment.

Flavonoid	Epigenetic Mechanisms	Renal Cancer Evidence	Preclinical/Clinical Evidence
Apigenin	Inhibits histone deacetilases HDAC1 and HDAC3; increases H3 acetylation at p21 promoter site; [170] influences miRNA and lncRNAs levels [171,172]	G2/M cell-cycle arrest, DNA damage, ATM signaling activation, and p53-dependent apoptosis [163]	Preclinical evidence
EGCG	Inhibits DNMTs, HDACs, and HATs; reverses promoter hypermethylation of tumor suppressors; modulates miRNAs and lncRNA levels [173]	MMP-2 and MMP-9 downregulation; activation of TFEB-mediated autophagy; inhibition of epithelial–mesenchymal transition [165,166]	Clinical studies of green tea extract as supportive therapy in prostate cancer [173]
Quercetin	Reduction of DNMT1/DNMT3a; inhibition of HDACs and HAT; effects on transcription co-activators and miRNA networks [174]	PI3K/AKT signaling and oxidative stress modulation [63]	Clinical trial NCT03476330 in Fanconi anemia-related squamous cell carcinoma [175]
Luteolin	Inhibits DNMTs, HDACs, HMTs, and HATs; affects histone methylation/acetylation; regulates miRNA/lncRNAs [176]	Inhibition of PI3K/AKT and MAPK signaling; ROS-mediated apoptosis [62]	Preclinical evidence
Genistein	Inhibits DNMTs; reduces global DNA methylation; modulates histone markers; affects miRNAs [177]	Downregulation of onco-miR-1260b and inhibition of Wnt-signaling [178]	Ongoing study in pediatric cancer patients: NCT02624388 [179]
Resveratrol	Induction of TP53, NRF2, and JDP2 axis in cancer cells to control ROS balance in mitochondria; modulation of HDACs (including SIRT1); downregulation of DNMT3b [180]	No RCC-specific studies	Clinical trials challenged by bioavailability/dosage issues, small or short-duration studies, heterogeneous formulations and randomization methods, clinical endpoints, and unassessed drug interactions [181]

Abbreviations: HDAC1 = Histone Deacetylase 1; HDAC3 = Histone Deacetylase 3; H3 = Histone H3; miRNA = MicroRNA; lncRNA = Long Non-Coding RNA; ATM = Ataxia-Telangiectasia Mutated; TP53 = Tumor Protein p53; DNMT = DNA Methyltransferase; DNMT1 = DNA Methyltransferase 1; DNMT3a = DNA Methyltransferase 3 Alpha; DNMT3b = DNA Methyltransferase 3 Beta; HAT = Histone Acetyltransferase; MMP-2 = Matrix Metalloproteinase 2; MMP-9 = Matrix Metalloproteinase 9; TFEB = Transcription Factor EB; PI3K = Phosphoinositide 3-Kinase; AKT = Protein Kinase B; HMT = Histone Methyltransferase; MAPK = Mitogen-Activated Protein Kinase; ROS = Reactive Oxygen Species; Wnt = Wingless/Integrated Signaling Pathway; SIRT1 = Sirtuin 1; NRF2 = Nuclear Factor Erythroid 2–Related Factor 2; JDP2 = Jun Dimerization Protein 2; RCC = Renal Cell Carcinoma.

## Data Availability

No new data were generated or analyzed in support of this research, as this study is a review article.
